# Pancreatic cancer incidence and mortality trends in urban Shanghai, China from 1973 to 2017: a joinpoint regression and age-period-cohort analysis

**DOI:** 10.3389/fonc.2023.1113301

**Published:** 2023-07-27

**Authors:** Mengyin Wu, Kai Gu, Yangming Gong, Chunxiao Wu, Yi Pang, Wei Zhang, Chunfang Wang, Yan Shi, Yingbin Liu, Chen Fu

**Affiliations:** ^1^ Division of Noncommunicable Diseases and Injury, Shanghai Municipal Center for Disease Control and Prevention, Shanghai, China; ^2^ SKLORG & Department of Epidemiology, Shanghai Cancer Institute, Renji Hospital, Shanghai Jiao Tong University School of Medicine, Shanghai, China; ^3^ Division of Public Health Informatics, Shanghai Municipal Center for Disease Control and Prevention, Shanghai, China; ^4^ Department of Biliary-Pancreatic Surgery, Renji Hospital Affiliated to Shanghai Jiao Tong University School of Medicine, Shanghai, China; ^5^ Shanghai Municipal Center for Disease Control and Prevention, Shanghai, China; ^6^ Shanghai Clinical Research Center for Aging and Medicine, Shanghai, China

**Keywords:** prevalence, trend, joinpoint regression analysis, age-period-cohort analysis, pancreatic cancer

## Abstract

**Background and purpose:**

To provide a comprehensive overview of epidemiological features and temporal trends of pancreatic cancer in urban Shanghai from 1973 to 2017.

**Methods:**

Data on pancreatic cancer in urban Shanghai were obtained through the Shanghai Cancer Registry and the Vital Statistics System. Joinpoint analysis was used to describe the temporal trends and annual percent changes (APCs) and age-period-cohort analysis were used to estimate the effects of age, period, and birth cohort on pancreatic cancer.

**Results:**

There were a total of 29,253 cases and 27,105 deaths of pancreatic cancer in urban Shanghai over the 45-year study period. The overall average annual age-standardized incidence and mortality rates were 5.45/100,000 and 5.02/100,000, respectively. Both the incidence and mortality rates demonstrated fluctuating upward trends, with an average annual increase rate of 1.51% (APC = 1.51, *P* < 0.001) and 1.04% (APC = 1.04, *P* < 0.001), respectively. The upward trend in incidence was greater for females than for males, while the trend in mortality was seen in both sexes equally and continuously. In recent years (2013-2017), the age-specific incidence rates increased further than before, with statistically significant changes in the 35-year, 45- to 55-year and 70- to 85-year age groups (*P* < 0.05). The age-specific mortality rates also showed obvious upward trends, which in the 50- to 55-year, and 75- to 85-year age groups increased significantly. The results of the age-period-cohort analysis suggested significant effects of age, period, and cohort on the prevalence of pancreatic cancer.

**Conclusion:**

The prevalence of pancreatic cancer, dramatically influenced by socioeconomic development and lifestyles, demonstrated a significant upward trend from 1973 to 2017 in urban Shanghai and underscored the necessity and urgency for additional efforts in primary and secondary prevention measures.

## 1 Introduction

Pancreatic cancer, with an extremely poor prognosis, is one of the most lethal malignancies. According to the latest estimates from GLOBOCAN 2020, there were a total of 496,000 new cases and 466,000 deaths of pancreatic cancer worldwide, and pancreatic cancer ranked 12^th^ in the incidence spectrum of malignancies but 7^th^ in the mortality spectrum (1). The incidence and mortality of pancreatic cancer vary greatly across regions and populations. Rates are higher in countries and regions with higher Human Development Index, such as Europe, North America, and Australia/New Zealand, and have remained relatively stable or slightly increased in recent years, which reflects the potential influence of lifestyles and consumption habits on the prevalence of pancreatic cancer (2, 3). The prevalence of pancreatic cancer in China is at the middle level in the world. Based on the *China Cancer Registry Annual Report 2019* by China National Cancer Center, there were 27,575 new cases of pancreatic cancer in China in 2016, and pancreatic cancer ranked as the 7^th^ most common cause of cancer deaths. The rates in China have been increased constantly in recent decades, and previous studies have suggested improvement in the diagnosis and some certain risk factors which have been identified, such as positive family history, genetics, diabetes, obesity, cigarette smoking, and alcohol drinking, may partly explain the rising trends (4–7).

Located on the eastern coast of China, Shanghai is an important economic and cultural center, which has been expanding rapidly in recent decades, and there have been dramatic changes in demographic structure and residents’ lifestyles. The epidemiological study of pancreatic cancer in urban Shanghai can expose the impact of urbanization and demographic changes on cancer prevalence trends, which is an important reference for cancer prevention and treatment in urban populations.

In this study, we examined the temporal trends on the age-standardized incidence and mortality rates of pancreatic cancer from 1973 to 2017 in urban Shanghai and quantified the effects of age, period, and birth cohort by using age-period-cohort analysis. Assessing the comprehensive estimates of the patterns and variation trends of pancreatic cancer can not only show the epidemiological characteristics and development trend of pancreatic cancer in urban Shanghai in detail but also provide clues to elucidate the etiology of pancreatic cancer and lay the foundation for the development of prevention strategies.

## 2 Materials and methods

### 2.1 Data sources

Data on pancreatic cancer in urban Shanghai from 1973 to 2017 were obtained through the population-based Shanghai Cancer Registry (SCR), an associate member of the International Association of Cancer Registries (IACR), and the Vital Statistics System of Shanghai Municipal Center for Disease Control and Prevention (Shanghai CDC), as previously described (8, 9). Briefly, the SCR systematically collects information on incident cancer cases in urban Shanghai, and complete incidence data have been available since 1973. The SCR has formed a standard system to collect, process, and report cancer incidence data. The corresponding population data of urban Shanghai were obtained from the demographic information regularly released by the Shanghai Public Security Bureau. We counted the number of pancreatic cancer cases and deaths, age-specific rates according to the year of diagnosis or death, sex, and age group, and used the world standard million population (Segi 1960) to calculate age-standardized rates of incidence and mortality (10), in which the age-specific rate was counted by grouping every 5-year group to 85 years and above, and the year of diagnosis or mortality was counted year by year or 5 years combined.

### 2.2 Statistical analysis

Temporal trend analysis was conducted by Joinpoint Trend Analysis Software 4.9 (https://surveillance.cancer.gov/joinpoint/) and the estimated annual percent changes (APCs) were used to represent the average percent increase or decrease in age-standardized rates per year of incidence and mortality on pancreatic cancer. Joinpoint trend analysis is a popular method that has been widely used to identify the best-fitting points (joinpoints), estimate the trends between joinpoints, and fit corresponding joined straight liens on a logarithmic scale to the trends in annual age-standardized rates (11). In this study, we set the allowed maximum number of joinpoints as 5 over 45 years, and at least 5 years was required for each segment.

Besides, we also conducted an age-period-cohort analysis to further examine the effects of age, period, and birth cohort on the temporal trends in age-standardized incidence and mortality rates of pancreatic cancer (12, 13). The age-period-cohort analysis was conducted by using the US NCI web-based statistical tool (https://analysistools.cancer.gov/apc/), according to the method proposed by Rosenberg et al. (14). For age-period-cohort analysis, we restricted the age of residents to 20-84 years to mitigate some of the variation and potential biases caused by few cases among the younger and the oldest age groups. We used the overall pancreatic cancer data by consecutive 5-year age groups (20-24, 25-29, …, 80-84), the same 5-year intervals for period cohorts (1973-1977, 1978-1982, …, 2013-2017) and birth cohorts (1893-1897, 1898-1902, …, 1993-1997). The mid-points of the period cohort (1993-1997) and birth cohort (1943-1947) were set as reference cohorts, respectively. Longitudinal age-specific rates, period and cohort ratio rates with corresponding 95% confidence intervals (RRs with 95% CIs), and local drifts with net drift were used to describe the temporal trends and effects of age, period, and birth cohort (15). Longitudinal age curves refer to the fitted longitudinal age-specific rates adjusting for period deviations in the reference cohort, the period effects refer to variations in age-standardized rates of pancreatic cancer in urban Shanghai over the whole study period simultaneously associating with all age groups. The cohort effects refer to changes in age-standardized rates across groups of individuals with the same birth years. Local drifts, generated from log-linear regressions, refer to the APCs in each age group. And net drift, the sum of the log-linear temporal trend arising from birth cohort effects, refers to the average APCs in age-standardized rates of birth. 1-*df* Wald test was used to identify the significance. We also conducted both analyses, the joinpoint analysis and the age-period-cohort analysis, by sex to examine the disparities in males and females. And statistical significance was attributed to two-sided *P*-values less than 0.05.

### 2.3 Availability of data and materials

Due to the grounds of our ethics approval, data from this study are unable to be shared. Most of the data supporting the conclusions of this study are available in the Cancer Incidence in Five Continents (CI5) series: Cancer Incidence in Five Continents Volumes I to X by IARC (http://ci5.iarc.fr/CI5I-X/Default.aspx).

### 2.4 Bioethics

This study has been reviewed and approved by the *Ethics Committee of Shanghai Municipal Center for Disease Control and Prevention*.

## 3 Results

**Figure 1:**
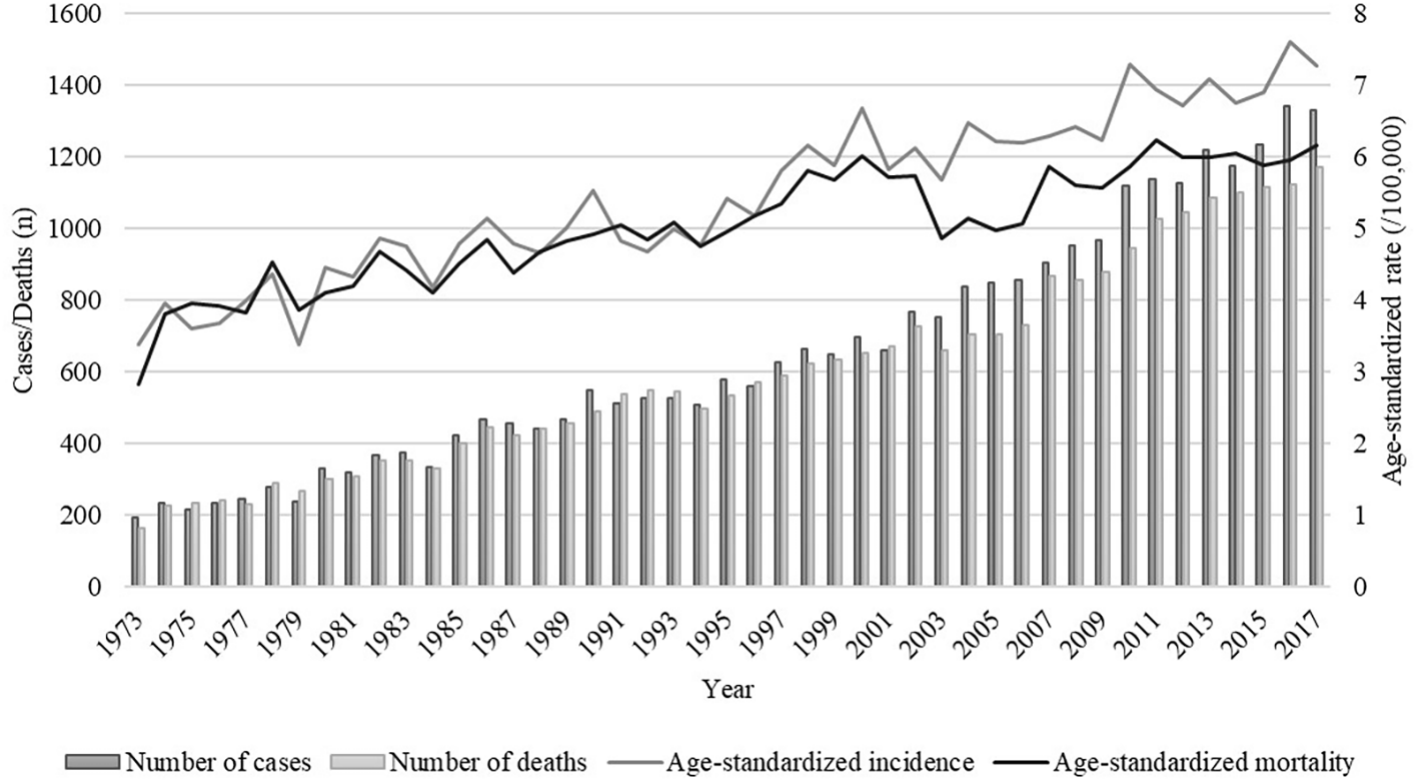
Trends of numbers and age-standardized rates of incidence and mortality of pancreatic cancer in urban Shanghai, 1973-2017.

Over the 45-year study period, there were a total of 29,253 cases (53.5% in males and 46.5% in females) and 27,105 deaths (53.2% in males and 46.8% in females) of pancreatic cancer among residents in urban Shanghai. As shown in **Figure 1**, both the age-standardized incidence and mortality rates of pancreatic cancer showed a fluctuating upward trend (age-standardized incidence rates ranged from 3.38/100,000 in 1973 to 7.28/100,000 in 2017 and age-standardized mortality rates ranged from 2.83/100,000 in 1973 to 6.17/100,000 in 2017), with the overall average annual age-standardized incidence and mortality rates were 5.45/100,000 and 5.02/100,000, respectively. The overall average annual age-standardized incidence and mortality rates of pancreatic cancer in males were 6.40/100,000 and 5.91/100,000, respectively, and those in females were 4.61/100,000 and 4.23/100,000, respectively (**Supplementary Figure 1**).

### 3.1 Joinpoint regression analysis

**Figure 2:**
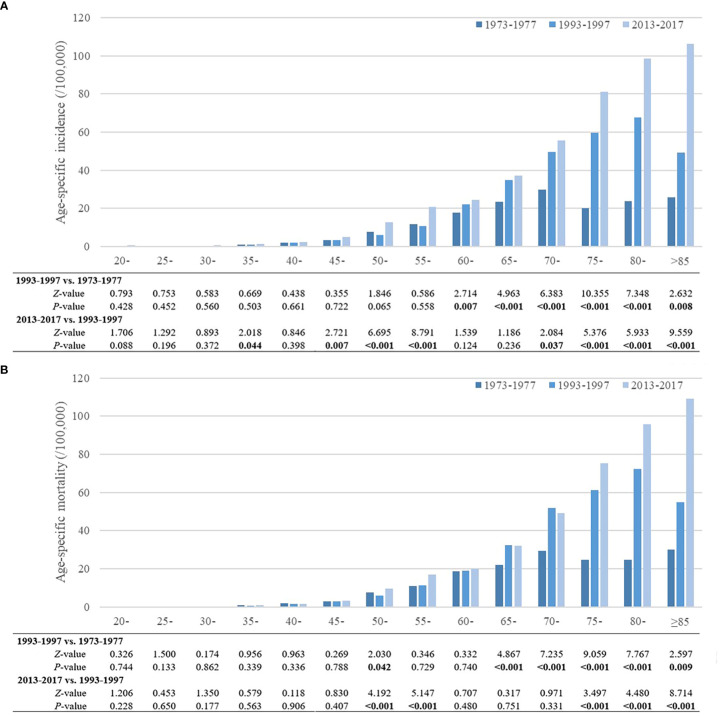
Trends of age-specific incidence and mortality rates of pancreatic cancer in urban Shanghai, 1973-2017. **(A)**. Trends of age-specific incidence rates of pancreatic cancer. **(B)**. Trends of age-specific mortality rates of pancreatic cancer.

The trends in age-specific incidence and mortality rates of pancreatic cancer in urban Shanghai during 1973-1977, 1993-1997, and 2013-2017 are shown in **Figure 2**. In general, the age-specific incidence and mortality rates of pancreatic cancer increased with age, and there has been an obvious increase in the age-specific incidence and mortality rates among the older adult population in recent years.

Compared to 1973-1977, the age-specific incidence rates of pancreatic cancer in urban Shanghai significantly increased during 1993-1997 for all residents aged 60 years and older (*P* < 0.05), while there were no significant differences for other age groups. During 2013-2017, the age-specific incidence rates increased further than before, with statistically significant changes in the 35-year, 45- to 55-year and 70- to 85-year age groups (*P* < 0.05). Similar to the age-specific incidence rates of pancreatic cancer, the age-specific mortality rates also showed an upward trend over the whole study period. Compared to 1973-1977, the age-specific mortality rates for residents in the 50-year age group and residents aged 65 years and older increased significantly during 1993-1997 (*P* < 0.05), and the age-specific mortality rates of residents in the 50- to 55-year, and 75- to 85-year age groups also increased further during 2013-2017. Trends in age-specific incidence and mortality rates of pancreatic cancer in urban Shanghai stratified by sex are shown in **Supplementary Tables 1, 2**, and the results among different sex subgroups were consistent with those in the total population.

**Figure 3:**
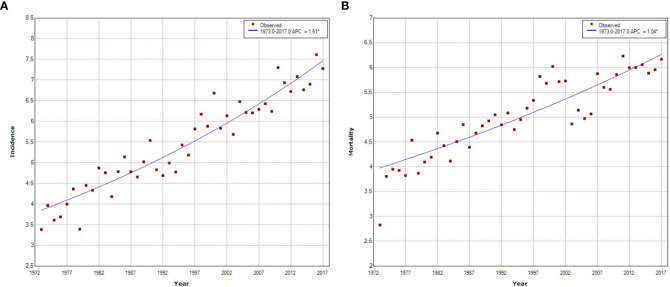
Joinpoint regression analysis of pancreatic cancer incidence and mortality trends in urban Shanghai, 1973–2017. **(A)**. Joinpoint regression analysis of pancreatic cancer incidence trends. **(B)**. Joinpoint regression analysis of pancreatic cancer mortality trends.

**Figure 3** shows the long-term trends and trend patterns for the age-standardized incidence and mortality rates of pancreatic cancer in urban Shanghai from 1973 to 2017 based on the joinpoint analyses. Both the age-standardized incidence and mortality rates demonstrated fluctuating upward trends during the whole study period, with an average annual increase rate of 1.51% (APC = 1.51, *P* < 0.001) and 1.04% (APC = 1.04, *P* < 0.001), respectively. The upward trend in age-standardized incidence was greater for females than for males (APC = 1.42, *P* < 0.001 for males vs. APC = 1.53, *P* < 0.001 for females, respectively), while the upward trend in age-standardized mortality was seen in both sexes equally and continuously during the entire studied period (APC = 0.99, *P* < 0.001 for males vs. APC = 0.96, *P* < 0.001 for females, respectively) (**Supplementary Figures 2, 3**).

### 3.2 Age−period−cohort analysis

**Figure 4:**
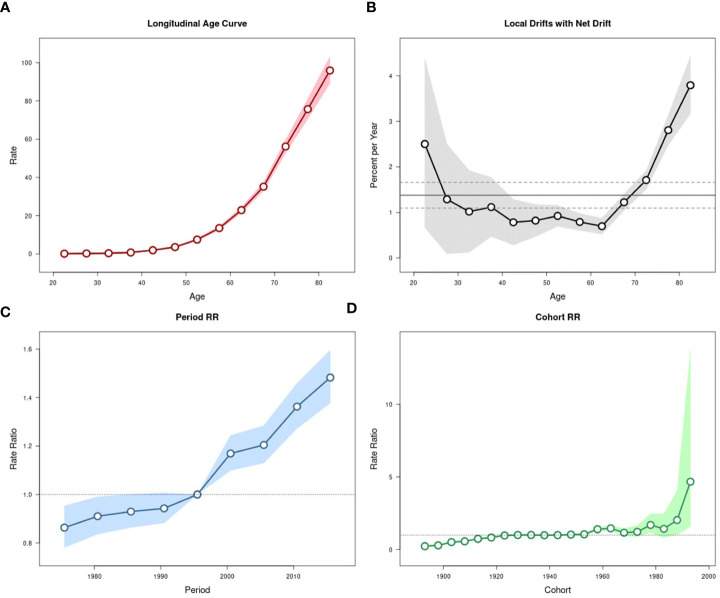
Estimated age-period-cohort effects for pancreatic cancer incidence in urban Shanghai during 1973-2017. **(A)**. Longitudinal age curve of pancreatic cancer incidence rates (/100,000 people) and corresponding 95% CIs. **(B)**. Local drift value for pancreatic cancer incidence rates: age group-specific annual percent change (%) in pancreatic cancer incidence rates and corresponding 95% CIs. **(C)**. Period effects on pancreatic cancer incidence rates: obtained from age period-cohort analyses for pancreatic cancer incidence rates and corresponding 95% CIs. **(D)**. Cohort effects on pancreatic cancer incidence rates: obtained from age-period-cohort analyses for pancreatic cancer incidence rates and corresponding 95% CIs.

**Figure 5:**
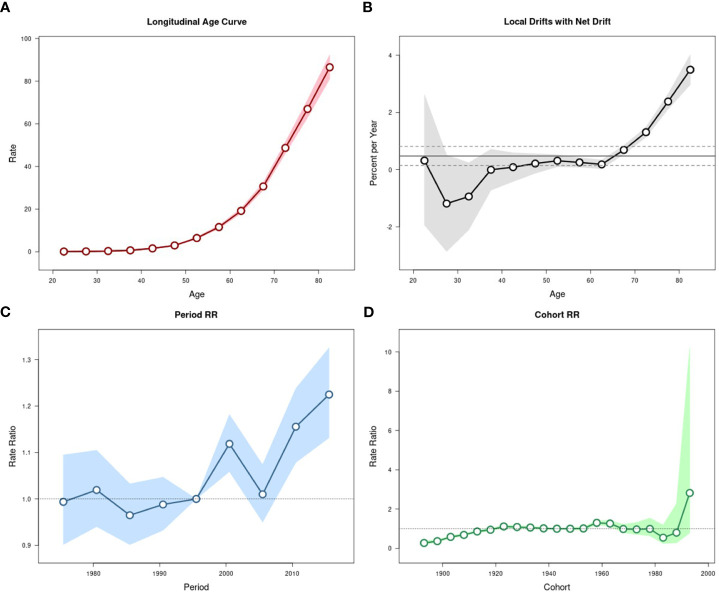
Estimated age-period-cohort effects for pancreatic cancer mortality in urban Shanghai during 1973-2017. **(A)**. Longitudinal age curve of pancreatic cancer mortality rates (/100,000 people) and corresponding 95% CIs. **(B)**. Local drift value for pancreatic cancer mortality rates: age group-specific annual percent change (%) in pancreatic cancer mortality rates and corresponding 95% CIs. **(C)**. Period effects on pancreatic cancer mortality rates: obtained from age period-cohort analyses for pancreatic cancer mortality rates and corresponding 95% CIs. **(D)**. Cohort effects on pancreatic cancer mortality rates: obtained from age-period-cohort analyses for pancreatic cancer mortality rates and corresponding 95% CIs.

The effects of age, period, and cohort on the age-standardized incidence and mortality rates of pancreatic cancer in urban Shanghai during 1973-2017 were shown in **Figures 4**, **5**, respectively.

The local drift values of age-standardized incidence rates fluctuated and decreased in all age groups below 60 years old, while significantly increasing among residents aged over 60 years. The period effects showed a continuous upward trend throughout the study period and increased at a greater rate in most recent years than in earlier years, with the corresponding RR in 2013-2017 being 1.48 (95% CI: 1.38-1.60). The cohort effects slowly increased from 1893 to 1923 (although all these RR values were less than 1), remained relatively stable and then fluctuated from 1928 to 1978, and showed a significant upward trend from 1983 to 1993, the corresponding RR for the cohort in 1993 is 4.67 (95% CI: 1.59-13.72). The results of the Wald test suggested statistically significant age, period, and cohort effects for age-standardized incidence, as did the local drifts and net drift (all *P*-values < 0.001).

The local drift values of age-standardized mortality rates showed a trend of fluctuation and decline with the increase of age in all age groups below 60 years old, while which showed an obvious upward trend among residents aged over 60 years, as did in age-standardized incidence rates. The period effects were significantly stronger in most recent years than in the early years but fluctuated somewhat in the middle of the study period, with the corresponding RR in 2013-2017 being 1.22 (95% CI: 1.13-1.34). The cohort effects on age-standardized mortality rates were similar to those on incidence rates, while there was a brief decrease before the upward trend from 1983 to 1993. The results of the Wald test suggested statistically significant age, period, and cohort effects for age-standardized mortality, as did the local drifts and net drift (*P*-values < 0.001 for age, period, and cohort effects, and local drifts, *P* = 0.005 for net drift).

The results of age-period-cohort analyses also suggested some differences in patterns of age, period, and cohort effects among different sex groups (**Supplementary Figures 4, 5** for age-standardized incidence rates and **Supplementary Figures 6, 7** for age-standardized mortality rates, respectively). The local drift values of age-standardized incidence rates fluctuated in age groups below 60 years old and showed an obvious upward trend in age groups over 60 years among males, which showed a U-shape trend with age among females. The trend of period effects on age-standardized incidence in males was similar to which in the total population. However, the period effects in females decreased slowly in the early years and increased rapidly from 1993 to 2017. The cohort effects in both sex groups were consistent with which in the total population. The results of Wald test suggested statistically significant age, period and cohort effects for age-standardized incidence, as did the local drifts and net drift for both sexes (all *P*-values < 0.001).

The local drift values of age-standardized mortality rates in males were significantly higher in the older adult group than in the younger group (except for the 20-year age group) but relatively flat in the 35- to 55-year age groups. The trend of local drift values in females was similar to which in the total population. The trend of period effects in males was consistent with those of the total population. However, the period effect fluctuated significantly in females, with the corresponding RR being 1.14 (95% CI: 0.98-1.32) from 1973 to 1977, higher than 1.11(95% CI: 0.98-1.25) from 2013 to 2017. The cohort effects were consistent with which on the total population in both sex groups. The results of the Wald test suggested statistically significant age, period, and cohort effects for age-standardized mortality, as did the local drifts and net drift for both sexes (all *P*-values < 0.001). However, the net drift was significant only in males (*P* = 0.004).

## 4 Discussion

The prevalent patterns of incidence and mortality rates of pancreatic cancer in urban Shanghai are closely related to the urbanization process and the change in population structure. Approximately 29,253 cases were diagnosed and 27,105 deaths died from pancreatic cancer in urban Shanghai over the 45-year study period. The results of trend analyses suggested that the age-standardized rates had obvious signs of increasing, with an average APC of 1.51% for incidence and 1.04% for mortality, respectively. Specifically, the trends were consistent with the general population across sex groups. However, the increase in either incidence or mortality was more pronounced in the older adults than in the younger.

The shifting age structure of the population with advanced imaging and expertise in pathology accounts for much of the increasing prevalence of pancreatic cancer, especially in high-income nations and regions (16). The past four decades have witnessed an over-doubling in the annual incidence and mortality rates of pancreatic cancer in urban Shanghai. As of 2017, the annual age-standardized rates were 7.28/100,000 and 6.17/100,000 for incidence and mortality, respectively, which were on par with other countries in East Asia and higher than the rest regions of China (1, 17). Either incidence or mortality rate for pancreatic cancer seems to be higher among males than in females worldwide (18). However, findings in this study suggested that the incidence in females have been increased faster than in males, noting that the potential burden for females suffering the risk from pancreatic cancer deserves further attention.

Regional and sex differences in the prevalence of pancreatic cancer have not been fully explained. In addition to inherited genetic and environmental factors, much of the increase is owing to the aging population worldwide, and there are key modifiable risk factors for pancreatic cancer, such as obesity, diabetes, cigarette smoking and alcohol consumption (19–21). A pooled analysis showed that subjects with an increased body mass index (BMI) suffered a 40% higher risk of pancreatic cancer compared with those who had a stable BMI, and the value of the waist-to-hip ratio was also positively associated with the risk of pancreatic cancer (22). Recent mendelian random analyses based on genome-wide data singled out BMI to be causally associated with an increased risk of pancreatic cancer (23, 24). Previous literature reported that pancreatic cancer might also be associated with diabetes and nearly 85% of patients had frank diabetes or impaired glucose tolerance (25). However, the relationship between pancreatic cancer and diabetes remains complex, given that both of those share common risk factors such as age, obesity, insulin resistance, and genetic factors et al, and the order of the progression of diabetes and pancreatic cancer is under indeterminate (26–28). A meta-analysis showed strong evidence that smoking was one of the major risk factors associated with pancreatic cancer, with an estimated population-attributable fraction of 11-32% (29). And another meta-analysis estimated an odds ratio of 1.74 (95% CI 1.61-1.87) for current smokers compared with non-smokers (30). Both results were consistent with findings presented in previous studies (31, 32), and given the diversity in smoking rates between males and females, this may partly explain the sex difference in pancreatic cancer prevalence. Alcohol consumption is also a well-established risk factor for pancreatic cancer (33, 34). However, the effect of alcohol consumption on pancreatic cancer among different sex groups remains uncertain. A previous study suggested that there was no significant interaction between alcohol consumption and sex (35). Another prospective study estimated a hazard ratio of 1.77 (95% CI 1.06-2.95) for the risk of pancreatic cancer in males who drank over 60 g alcohol per day compared with moderate drinkers who drank 0.1-4.9 g alcohol per day, while the association was not significant in females who drank over 30 g alcohol per day (36). According to the *Shanghai Non-communicable and Chronic Disease Surveillance Report* from Shanghai CDC, the alcohol consumption rate of males in Shanghai was 13.07%, 39.97%, and 45.01% in 2007, 2013, and 2017, respectively. For females, the alcohol consumption rate was 0.82%, 8.49%, and 16.47%, respectively. This may contribute to an increased risk for females to suffer from pancreatic cancer.

This study suggested that the prevalence of pancreatic cancer in urban Shanghai was mainly concentrated in residents aged 50 years and above, which was consistent with the results of previous studies (19, 37). However, the youthful trend of pancreatic cancer prevalence cannot be ignored (38). In recent years, the age-specific incidence rates of some age groups under 50 years old increased significantly in urban Shanghai. The increased exposure of chronic disease risk factors to residents and the increased number of chronic disease patients may lead to an upward trend of pancreatic cancer prevalence. A multi-center case-control study in China also revealed the association between various risk factors for chronic diseases and pancreatic cancer, which was also consistent with a previous case-control study in Shanghai and the trends we observed (39).

The strength of this study is that it provided an up-to-date and comprehensive overview of temporal trends and estimates of age-period-cohort analysis on pancreatic cancer in urban Shanghai from 1973-2017. The data source of this study was reliable and the study population was representative of Asian residents, which can further supplement the lack of research on pancreatic cancer in Asia. However, there were still several limitations in this study. First, this study was an observational study and did not explore the effects of possible risk factors on pancreatic cancer due to the limitation of data. Second, the study area was limited to Shanghai, further studies are suggested to be performed in other regions of Asia to fully elucidate potential risk factors and regional differences for pancreatic cancer.

## 5 Conclusion

In summary, the overall age-standardized incidence and mortality rates of pancreatic cancer demonstrated significant upward trends from 1973 to 2017 in urban Shanghai. The trends among different sexes were consistent with those in the total population and the increases in rates were more pronounced in the older adults than in the younger. The results of the age-period-cohort analysis also suggested a dramatic influence of socioeconomic development and lifestyles on the prevalence of pancreatic cancer in urban shanghai over the past several decades and underscored the necessity and urgency for additional efforts in primary and secondary prevention measures.

## Data availability statement

The datasets presented in this article are not readily available because. Due to the grounds of our ethics approval, data from this study are unable to be shared. Most of data supporting the conclusions of this study is available in the Cancer Incidence in Five Continents (CI5) series: Cancer Incidence in Five Continents Volumes I to X by IARC (http://ci5.iarc.fr/CI5I-X/Default.aspx). Requests to access the datasets should be directed to http://ci5.iarc.fr/CI5I-X/Default.aspx.

## Ethics statement

The studies involving human participants were reviewed and approved by Ethic Committee of Shanghai Municipal Center for Disease Control and Prevention. Written informed consent for participation was not required for this study in accordance with the national legislation and the institutional requirements.

## Author contributions

MW, KG and YG designed the study. CXW, YP, WZ and CFW selected and processed the data. MW, KG and YG edited and revised the manuscript. YS, YL and CF provided useful information. All authors contributed to the subsequent drafts. The authors reviewed and endorsed the final submission.
