# Supplementary material for: Pancreatic cancer incidence and mortality trends in urban Shanghai, China from 1973 to 2017: a joinpoint regression and age-period-cohort analysis
Source: Front Oncol. 2023 Jul 27;13:1113301. doi: 10.3389/fonc.2023.1113301 (PMC10414985; doi:10.3389/fonc.2023.1113301)
Supplement: Supplementary file 1 [file DataSheet_1.pdf]

Supplementary materials

Pancreatic cancer incidence and mortality trends in urban Shanghai, China from 1973 to 2017: a joinpoint regression and age-period-cohort analysis

Figure 1. Trends of numbers and age-standardized incidence and mortality rates of pancreatic cancer by sex in urban Shanghai, 1973-2017

Table 1. Trends of age-specific incidence rates of pancreatic cancer stratified by sex in urban Shanghai, 1973-2017

Table 2. Trends of age-specific mortality rates of pancreatic cancer stratified by sex in urban Shanghai, 1973-2017

Figure 2. Joinpoint regression analysis of pancreatic cancer incidence trends stratified by sex in urban Shanghai, 1973–2017

Figure 3. Joinpoint regression analysis of pancreatic cancer mortality trends stratified by sex in urban Shanghai, 1973–2017

Figure 4. Estimated age-period-cohort effects for pancreatic cancer incidence in urban Shanghai during 1973-2017 among male

Figure 5. Estimated age-period-cohort effects for pancreatic cancer incidence in urban Shanghai during 1973-2017 among female

Figure 6. Estimated age-period-cohort effects for pancreatic cancer mortality in urban Shanghai during 1973-2017 among male

Figure 7. Estimated age-period-cohort effects for pancreatic cancer mortality in urban Shanghai during 1973-2017 among female

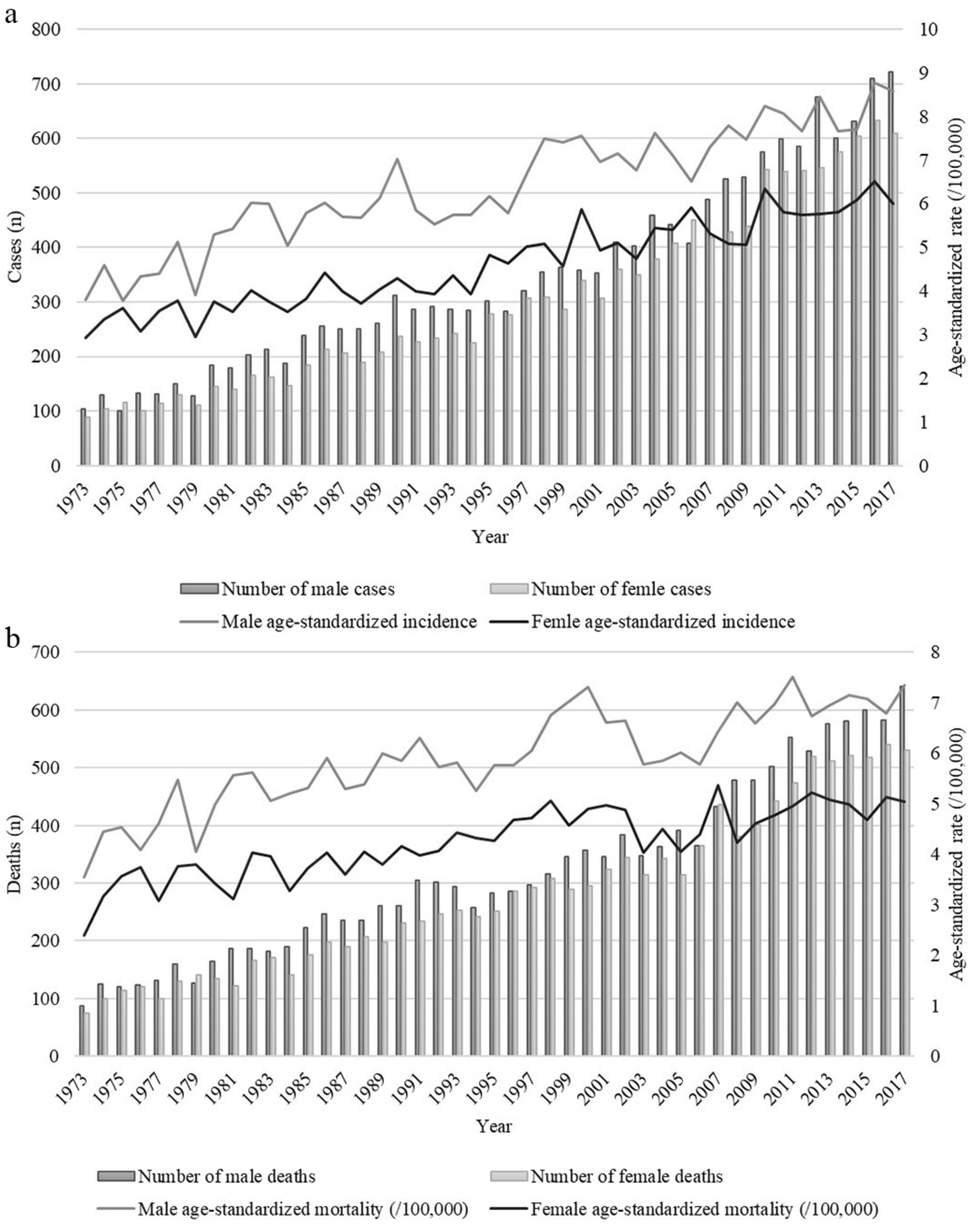

Figure 1. Trends of numbers and age-standardized incidence and mortality rates of pancreatic cancer by sex in urban Shanghai, 1973-2017

Table 1. Trends of age-specific incidence rates of pancreatic cancer stratified by sex in urban Shanghai, 1973-2017

	Age Group	Age-specific rate/100,000			Age-specific rate 1993-1997/1973-1977			Age-specific rate 2017-2013/1993-1997		
		**1973-1977**	**1993-1997**	**2013-2017**	**Rate**	***Z*-value**	***P*-value**	**Rate**	***Z*-value**	***P*-value**
**Male**	20-	0.08	0.11	0.47	1.31	0.190	0.849	4.48	1.254	0.210
	25-	0.33	0.10	0.27	0.30	1.168	0.243	2.72	0.733	0.464
	30-	0.24	0.48	0.56	1.96	0.530	0.596	1.17	0.171	0.864
	35-	1.40	0.98	1.89	0.70	0.864	0.387	1.92	1.364	0.172
	40-	1.90	2.57	3.00	1.35	1.020	0.308	1.17	0.420	0.675
	45-	3.14	3.68	7.12	1.17	0.467	0.641	1.94	2.697	**0.007**
	50-	9.45	6.28	16.29	0.66	1.995	**0.046**	2.60	6.703	**<0.001**
	55-	12.98	13.20	26.30	1.02	0.095	0.924	1.99	6.997	**<0.001**
	60-	20.72	26.75	29.98	1.29	1.969	**0.049**	1.12	1.240	0.215
	65-	25.02	40.06	41.89	1.60	3.392	**0.001**	1.05	0.474	0.636
	70-	34.24	52.81	58.40	1.54	3.158	**0.002**	1.11	1.051	0.293
	75-	28.28	62.31	85.93	2.20	3.390	**0.001**	1.38	3.635	**<0.001**
	80-	32.67	80.20	103.64	2.46	3.961	**<0.001**	1.29	2.550	**0.011**
	≥85	9.62	68.94	107.94	7.17	5.836	**<0.001**	1.57	3.836	**<0.001**
**Female**	20-	0.00	0.12	0.46	-	1.000	0.317	3.73	1.150	0.250
	25-	0.19	0.23	0.46	1.19	0.145	0.884	2.01	0.905	0.366
	30-	0.27	0.22	0.48	0.82	0.162	0.871	2.14	0.750	0.453
	35-	0.60	0.63	0.99	1.06	0.070	0.944	1.58	0.805	0.421
	40-	2.08	1.00	1.44	0.48	1.723	0.085	1.44	0.664	0.506
	45-	3.60	2.63	3.01	0.73	1.027	0.304	1.15	0.391	0.696
	50-	6.22	6.04	8.84	0.97	0.120	0.905	1.46	2.170	**0.030**
	55-	10.21	8.51	15.52	0.83	0.877	0.380	1.82	4.703	**<0.001**
	60-	14.67	17.94	18.72	1.22	1.228	0.219	1.04	0.353	0.724
	65-	22.31	29.95	32.03	1.34	2.188	**0.029**	1.07	0.591	0.555
	70-	26.14	46.58	52.81	1.78	5.224	**<0.001**	1.13	1.330	0.184
	75-	15.29	57.45	77.00	3.76	7.065	**<0.001**	1.34	3.601	**<0.001**
	80-	19.94	59.46	94.92	2.98	3.760	**<0.001**	1.60	5.260	**<0.001**
	≥85	30.55	40.18	105.47	1.32	1.824	0.068	2.63	12.376	**<0.001**

Table 2. Trends of age-specific mortality rates of pancreatic cancer stratified by sex in urban Shanghai, 1973-2017

	Age Group	Age-specific rate/100,000			Age-specific rate 1993-1997/1973-1977			Age-specific rate 2017-2013/1993-1997		
		1973-1977	1993-1997	2013-2017	Rate	*Z*-value	*P*-value	Rate	*Z*-value	*P*-value
**Male**	20-	0.16	0.00	0.31	0.00	1.414	0.157	-	1.414	0.157
	25-	0.33	0.10	0.00	0.30	1.168	0.243	0.00	0.779	0.436
	30-	0.24	0.41	0.08	1.68	0.429	0.668	0.20	0.822	0.411
	35-	1.05	0.69	1.26	0.66	0.830	0.406	1.83	1.031	0.303
	40-	1.61	2.19	2.09	1.36	0.992	0.321	0.95	0.121	0.904
	45-	2.40	3.28	4.74	1.37	0.799	0.424	1.44	1.291	0.197
	50-	8.92	5.27	12.42	0.59	2.430	**0.015**	2.36	5.175	**<0.001**
	55-	11.76	13.33	22.73	1.13	0.640	0.522	1.70	5.203	**<0.001**
	60-	23.28	22.62	25.42	0.97	0.229	0.819	1.12	1.162	0.245
	65-	23.00	37.25	36.59	1.62	3.176	**0.001**	0.98	0.181	0.857
	70-	36.33	54.26	52.15	1.49	3.038	**0.002**	0.96	0.402	0.688
	75-	28.28	64.70	81.35	2.29	3.319	**0.001**	1.26	2.560	**0.010**
	80-	40.21	85.33	100.68	2.12	2.475	**0.013**	1.18	1.632	0.103
	≥85	28.86	71.94	110.08	2.49	4.149	**<0.001**	1.53	3.672	**<0.001**
**Female**	20-	0.00	0.12	0.15	-	1.000	0.317	1.24	0.153	0.878
	25-	0.10	0.00	0.18	0.00	0.498	0.619	-	1.618	0.106
	30-	0.27	0.15	0.16	0.55	0.427	0.669	1.07	0.061	0.952
	35-	0.60	0.47	0.20	0.79	0.268	0.788	0.42	0.728	0.467
	40-	1.99	0.65	0.96	0.33	2.305	**0.021**	1.48	0.634	0.526
	45-	3.23	2.02	1.63	0.62	1.325	0.185	0.81	0.454	0.650
	50-	6.22	6.31	6.42	1.01	0.063	0.950	1.02	0.091	0.928
	55-	9.81	9.05	11.04	0.92	0.395	0.693	1.22	1.387	0.165
	60-	13.97	15.79	14.58	1.13	0.714	0.475	0.92	0.587	0.557
	65-	20.93	28.13	26.90	1.34	2.150	**0.032**	0.96	0.369	0.712
	70-	23.85	49.86	45.93	2.09	5.856	**<0.001**	0.92	0.855	0.392
	75-	22.68	58.63	69.78	2.59	6.218	**<0.001**	1.19	2.059	**0.039**
	80-	17.72	63.34	92.24	3.57	4.307	**<0.001**	1.46	4.183	**<0.001**
	≥85	30.55	47.11	108.67	1.54	2.898	**0.004**	2.31	10.776	**<0.001**

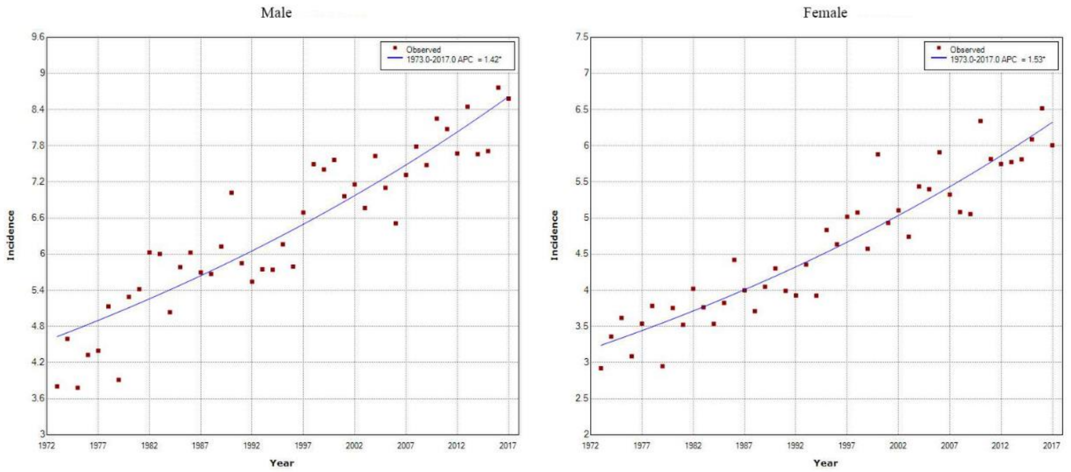

Figure 2. Joinpoint regression analysis of pancreatic cancer incidence trends stratified by sex in urban Shanghai, 1973–2017 (a. Joinpoint regression analysis of pancreatic cancer incidence trends among male. b. Joinpoint regression analysis of pancreatic cancer incidence trends among female.)

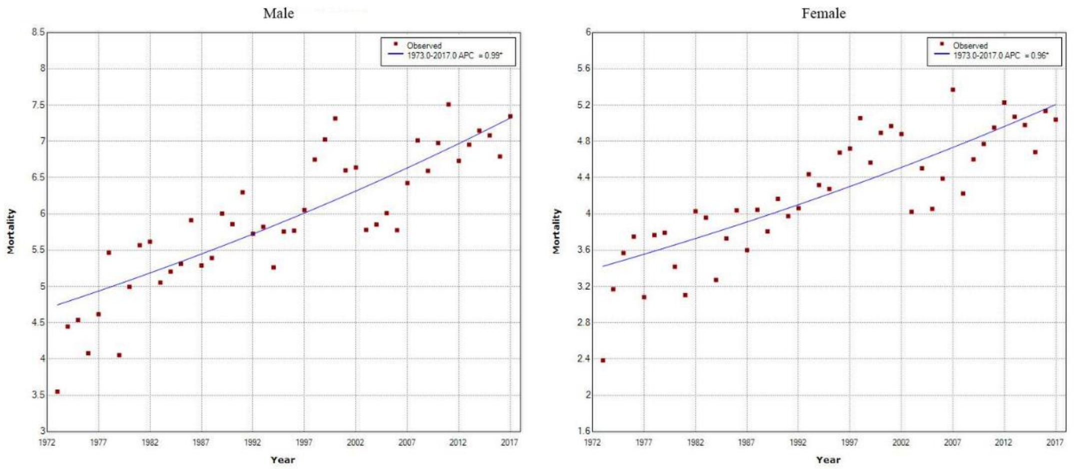

Figure 3. Joinpoint regression analysis of pancreatic cancer mortality trends stratified by sex in urban Shanghai, 1973–2017 (a. Joinpoint regression analysis of pancreatic cancer mortality trends among male. b. Joinpoint regression analysis of pancreatic cancer mortality trends among female.)

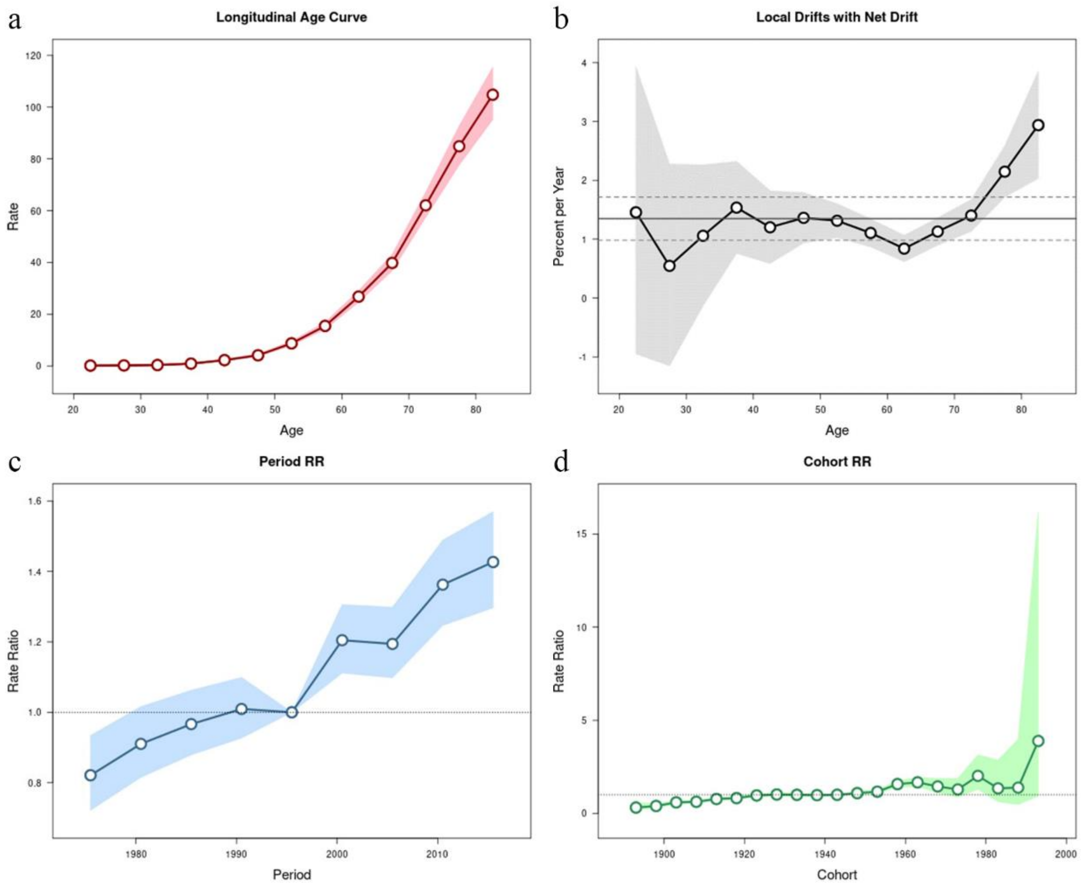

Figure 4. Estimated age-period-cohort effects for pancreatic cancer incidence in urban Shanghai during 1973-2017 among male (a. Longitudinal age curve of pancreatic cancer incidence rates (/100,000 people) and corresponding 95% CIs. b. Local drift value for pancreatic cancer incidence rates: age group-specific annual percent change (%) in pancreatic cancer incidence rates and corresponding 95% CIs. c. Period effects on pancreatic cancer incidence rates: obtained from age period-cohort analyses for pancreatic cancer incidence rates and corresponding 95% CIs. d. Cohort effects on pancreatic cancer incidence rates: obtained from age-period-cohort analyses for pancreatic cancer incidence rates and corresponding 95% CIs.)

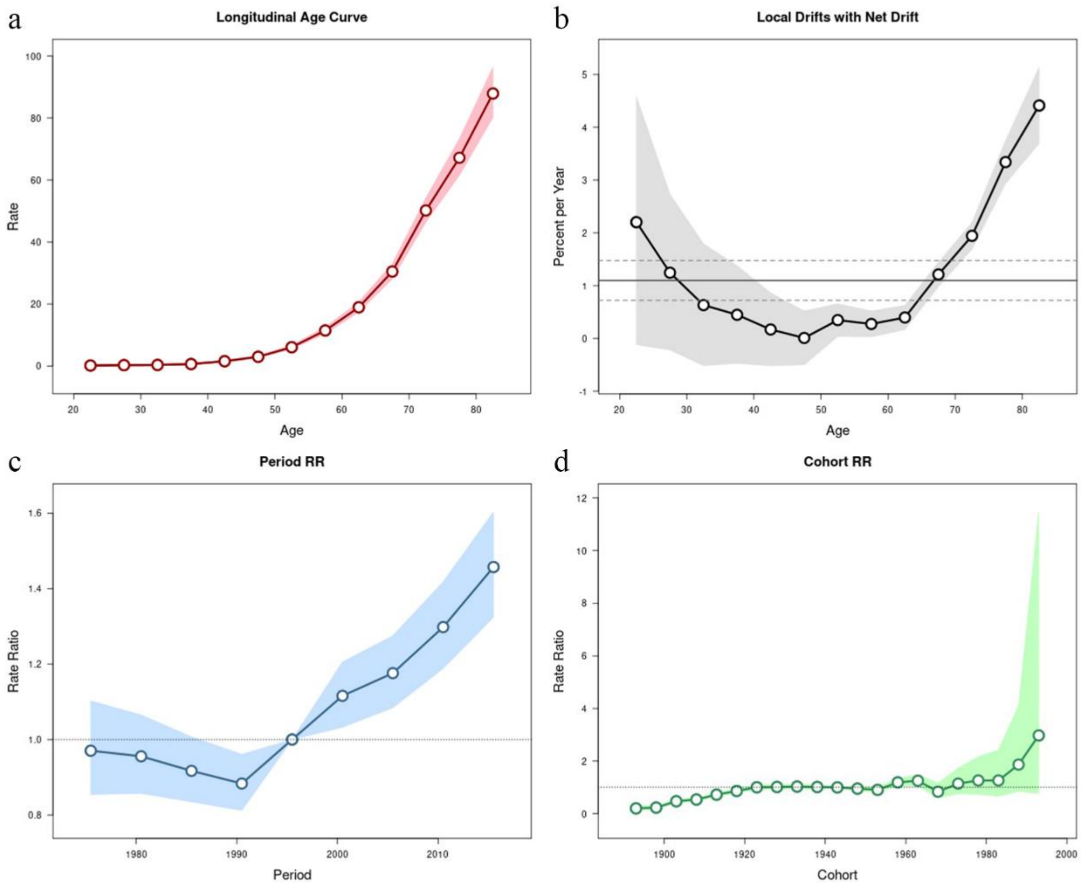

Figure 5. Estimated age-period-cohort effects for pancreatic cancer incidence in urban Shanghai during 1973-2017 among female (a. Longitudinal age curve of pancreatic cancer incidence rates (/100,000 people) and corresponding 95% CIs. b. Local drift value for pancreatic cancer incidence rates: age group-specific annual percent change (%) in pancreatic cancer incidence rates and corresponding 95% CIs. c. Period effects on pancreatic cancer incidence rates: obtained from age period-cohort analyses for pancreatic cancer incidence rates and corresponding 95% CIs. d. Cohort effects on pancreatic cancer incidence rates: obtained from age-period-cohort analyses for pancreatic cancer incidence rates and corresponding 95% CIs.)

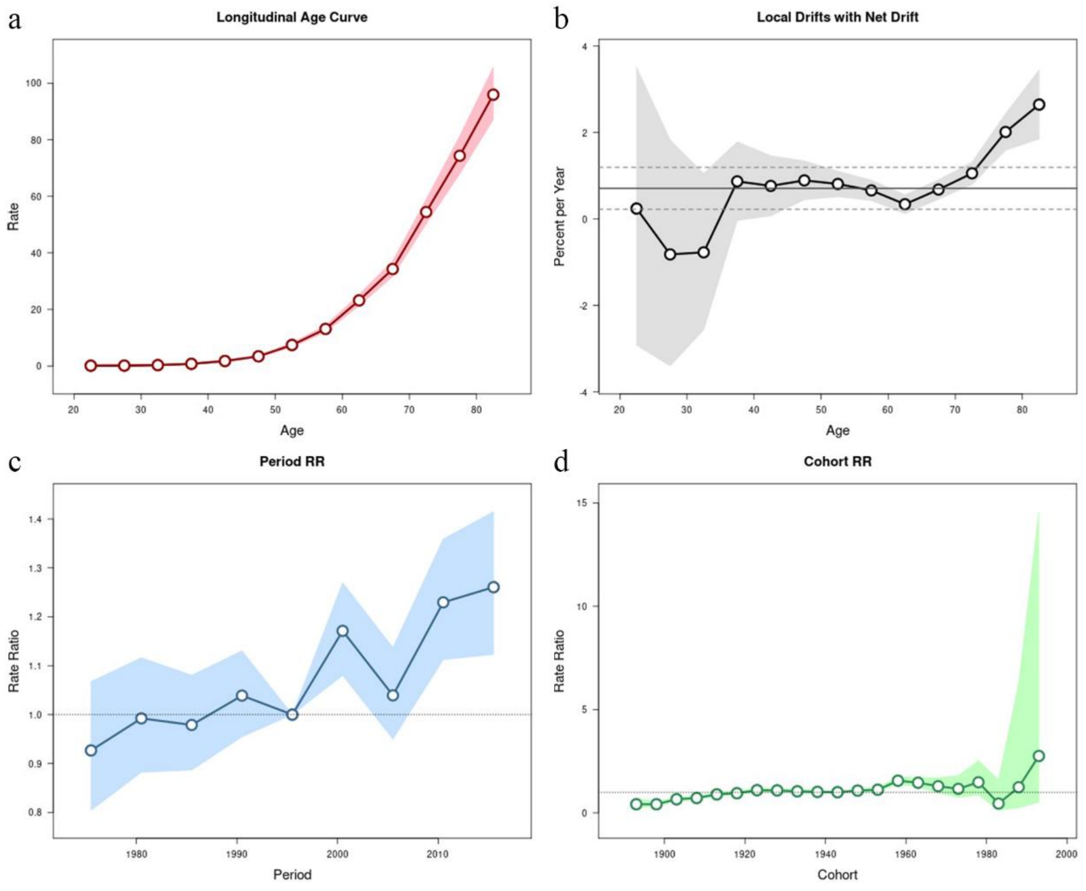

Figure 6. Estimated age-period-cohort effects for pancreatic cancer mortality in urban Shanghai during 1973-2017 among male (a. Longitudinal age curve of pancreatic cancer mortality rates (/100,000 people) and corresponding 95% CIs. b. Local drift value for pancreatic cancer mortality rates: age group-specific annual percent change (%) in pancreatic cancer mortality rates and corresponding 95% CIs. c. Period effects on pancreatic cancer mortality rates: obtained from age period-cohort analyses for pancreatic cancer mortality rates and corresponding 95% CIs. d. Cohort effects on pancreatic cancer mortality rates: obtained from age-period-cohort analyses for pancreatic cancer mortality rates and corresponding 95% CIs.)

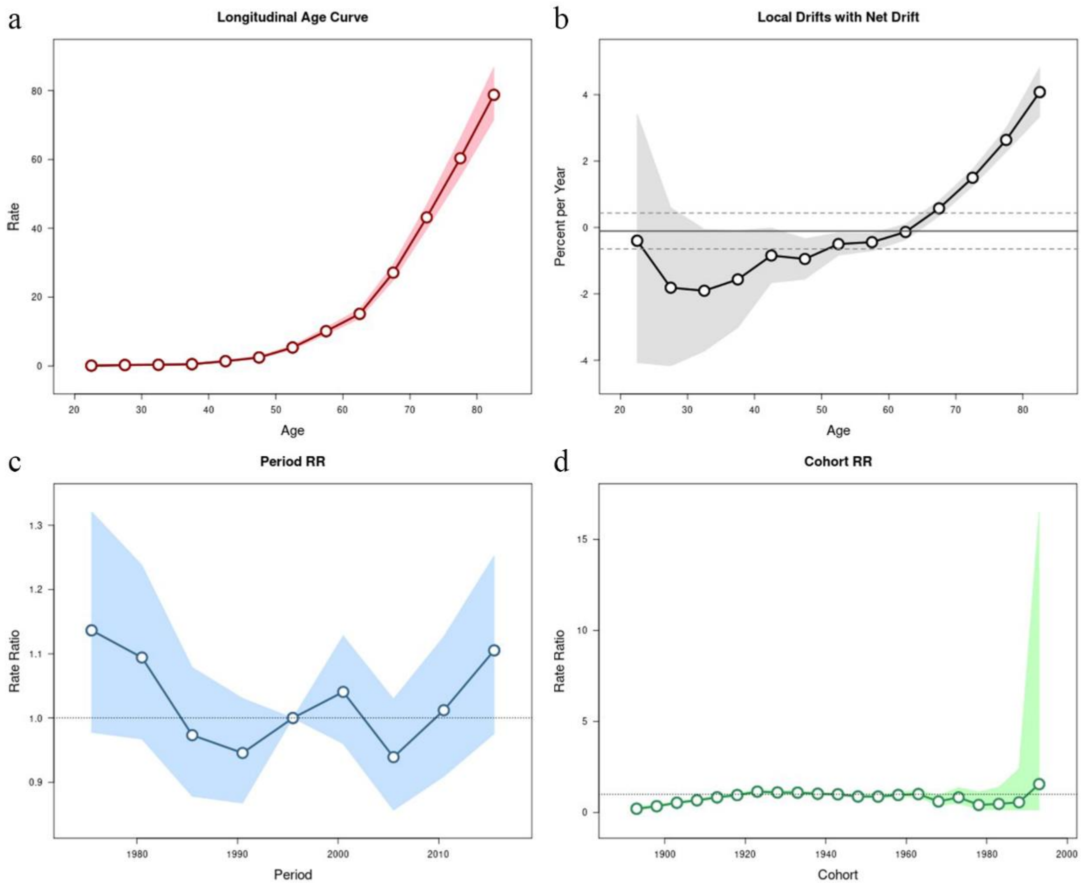

Figure 7. Estimated age-period-cohort effects for pancreatic cancer mortality in urban Shanghai during 1973-2017 among female (a. Longitudinal age curve of pancreatic cancer mortality rates (/100,000 people) and corresponding 95% CIs. b. Local drift value for pancreatic cancer mortality rates: age group-specific annual percent change (%) in pancreatic cancer mortality rates and corresponding 95% CIs. c. Period effects on pancreatic cancer mortality rates: obtained from age period-cohort analyses for pancreatic cancer mortality rates and corresponding 95% CIs. d. Cohort effects on pancreatic cancer mortality rates: obtained from age-period-cohort analyses for pancreatic cancer mortality rates and corresponding 95% CIs.)
